# Designing Junior Sport to Maximize Potential: The Knowns, Unknowns, and Paradoxes of Scaling Sport

**DOI:** 10.3389/fpsyg.2019.02878

**Published:** 2020-01-08

**Authors:** Tim Buszard, Damian Farrow, Machar Reid

**Affiliations:** ^1^Institute for Health and Sport (iHeS), Victoria University, Melbourne, VIC, Australia; ^2^Game Insight Group (GIG), Tennis Australia, Melbourne, VIC, Australia

**Keywords:** modified sport, scaling task constraints, ecological dynamics, junior sport, skill acquisition, children’s sport

## Abstract

In this mini-review, we draw attention to an important yet relatively untapped topic in the developmental pathway – the design of junior sport so that it appropriately matches the functional capacities of children. Junior sport is a regular weekend activity for many children across the world, yet many will be required to prematurely play on a field or with equipment that is designed for adults. Herein lies an opportunity for sport administrators to nurture children’s development in sport by appropriately manipulating the rules and dimensions of the game. The aim of this mini-review is to (1) draw attention to the value of scaling junior sport, (2) highlight paradoxes within the current scaling sport literature, and (3) emphasize a way forward for junior sport research. If we are genuine in our endeavor to tailor sports experiences for children, more sophisticated approaches to scaling those experiences are a must.

## Introduction

Junior sport is a regular weekend activity for many children across the world. It forms part of contemporary junior sport participation products or programs, often targeted at children aged 10 U, or features as the (only) pathway for retaining children in the game whom are transitioning out of these junior sport products. Regardless of an individual child’s introduction to sport, many will be required to prematurely play on a field or with equipment that is designed for adults. Should we expect the 10-year-old tennis player to experience success and remain in the sport when they cannot serve the ball into the service box on a full-sized court? What about the talented 12-year-old cricket player, who each week faces bowlers who find it difficult to land the ball on a full-sized pitch? Are these environments or experiences maximizing skill learning and engendering a love of the game?

Scaling sport is often perceived as merely an entry-level strategy to attract children into sports. As alluded to above, certainly there are examples of sports across the world that have grown their participation base on the back of modified programs that allow children to play the game with greater ease. However, at least three glaring issues remain with the design and implementation of modified junior sport: first, the rate at which its features or task constraints (ball size, field size, etc.) scale to the adult game is crude and arbitrary; second, many junior sport programs are facilitated or led by *coaches* while competitions are not; and third, a child’s exposure to sport in *what might be considered more appropriate* modified conditions is limited. Indeed, our systematic review highlighted that many sports require children to play in adult environments by the age of 10 (Buszard et al., 2016). Perhaps unsurprisingly, this coincides with a high dropout from sport during pre-adolescent years. For example, a longitudinal study of modified sport in Australia found that fewer than 25% of girls and 14% of boys transitioned from a (coach-led) modified sport program, designed for children aged 5–10 years, to club competitions that target players from 10 years of age (Eime et al., 2015). With this in mind, it is possible that sport authorities could better transition children into full-sized conditions by better scaling competition for some age groups and/or skill levels.

In this article, we critique empirical evidence that highlights the effect of scaled environments in junior sport. Indeed, the purpose of this mini-review is to draw attention to the potential value of scaling junior sport, while simultaneously highlighting the unknowns and paradoxes within the scaling sport literature. Although we recently published a systematic review on scaling junior sport (Buszard et al., 2016), we felt that it was important to highlight key issues to be addressed (that were not discussed in the systematic review) given the attention that is being given to modified sport within sport organizations. It should become clear that more sophisticated approaches to scaling junior sport are necessary if we are genuine in our endeavor to tailor sports experiences for children.

## What Do We Know About Scaling?

### A Brief Recap of the Scaling Literature

Scaling junior sport is not a new concept, but it has become popular over the past decade. Research began focusing on scaling in the 1960s (Wright, 1967), albeit in small doses (Elliott, 1981; Satern et al., 1989; Regimbal et al., 1992). It was not until the rise of formalized modified sport programs, which have predominately been developed based on intuitive reasoning rather than empirical evidence, that we have seen an exponential increase in the number of studies investigating scaling sport. By way of example, the International Tennis Federation’s launch of the ITF Tennis Play and Stay Campaign – its modified tennis for children aged 5–10 years – was the impetus for Hammond and Smith’s (2006) investigation of lower compression tennis balls for children aged 11 years and younger. This subsequently led to Farrow and Reid’s (2010) study on ball compression and court size, and since then sport scientists and academics have examined its use in a range of sports, including tennis (e.g., Buszard et al., 2014a; Kachel et al., 2015; Timmerman et al., 2015; Fitzpatrick et al., 2017), basketball (e.g., Arias et al., 2012a; Arias-Estero and Cánovas, 2014; Arias-Estero et al., 2018), cricket (Harwood et al., 2018a,b, 2019), Australian football (Hadlow et al., 2017), and soccer (Sarmento et al., 2018). The following sections describe what we have learnt about scaling junior sport.

### Theoretical Underpinnings

The rationale for scaling sport is predominantly underpinned by an ecological dynamics viewpoint of human movement and skill acquisition. Ecological dynamics advocates that an individual’s behavior emerges from the self-organization of perception and action under interacting constraints (organismic, environmental, and task) (Araujo et al., 2006; Davids et al., 2012). Indeed, the constraints imposed by sport (e.g., field size, number of players, equipment, duration of match, etc.) determine the boundaries of what actions are possible (referred to as the *affordance landscape*) (Newell, 1986). Clever manipulation of constraints can therefore influence the emergence of skills and strategies that children can learn while maintaining information-movement couplings that are inherent to the sport (Fitzpatrick et al., 2018). Hence, by altering the constraints of a game (e.g., smaller tennis court) to match the functional capacities of the individual (e.g., small stature and minimal strength), the task is simplified and children are able to perform skills that are otherwise not possible (e.g., explore depth and width of the court with groundstrokes). Accordingly premise of scaling sport is to augment the development of skills that are considered desirable for long-term performance and retention in the sport.

Scaling sport also has theoretical support from the theory of implicit motor learning. Implicit motor learning contends that learning is more durable and robust when the learner acquires a skill without conscious awareness of the underlying mechanics (Masters, 1992). Often motor skills are learnt implicitly when errors have been reduced (referred to as *error reduced learning*) as this limits the need for the learner to consciously analyze and correct errors (Maxwell et al., 2001). Given that a key benefit of scaling sport is that it heightens success for children (Buszard et al., 2014a), it is reasoned that appropriate scaling will reduce any tendency for children to correct errors, thereby evoking a more implicit mode of learning. We attempted to test this hypothesis by assessing the acute effect of equipment on children’s ability to perform a tennis task under dual-task conditions (Buszard et al., 2014b). We found that lesser skilled children performed significantly poorer under dual-task conditions when using full-sized but not scaled equipment. This suggested that there was greater conscious involvement when using inappropriately sized equipment. Indeed, this provided initial support that scaling equipment might promote implicit motor learning, but a learning study is required to consolidate this view.

### The Junior Sport Experience in a Scaled Environment

A number of studies have examined the effect of scaling junior sport by assessing performance in competitive matches. These studies have typically compared performance in scaled environments with performance in full-size environments. Children who are beginners to tennis created longer rallies and experienced more success with serving when playing with lower compression balls on smaller courts (Fitzpatrick et al., 2017). Lower compression balls as well as lower net heights have also positively affected the competition experience of highly talented children with more approaches to the net, more winners, and more successful serving (Kachel et al., 2015; Timmerman et al., 2015; Limpens et al., 2018). Similar themes have emerged in basketball, where a lighter ball has resulted in more dribbling and passing (Arias et al., 2012a), increased shot frequency and greater shot success (Arias et al., 2012a,b), a higher percentage of attempted lay-ups (Arias, 2012a), and more one-on-one situations (Arias et al., 2012c). Ultimately, when we modify or scale the constraints of junior sport, we are shaping children’s sporting experience, and we are facilitating exploration. Certainly there is strong evidence to show that appropriate modifications can facilitate rather than confine the emergence of desirable actions and behaviors for children playing sport.

### Skill Acquisition

Appropriate scaling of task constraints can facilitate the emergence of more desirable movement patterns. For example, beginner tennis players strike the ball more often with a low to high swing when using low compression balls compared to standard balls (Buszard et al., 2014a). Likewise, in cricket, 13-year-old fast bowlers displayed less shoulder counter rotation when bowling on shorter pitch lengths (Elliott et al., 2005). Significantly, shoulder counter rotation is linked with low back stress fractures – a common injury in junior fast bowlers (Elliott, 2000). In these examples, the assumption is that these coordinative patterns will become stable movement solutions with enough repetition. From a decision-making perspective, a shorter pitch length in cricket also increases the likelihood that children will make decisions when batting that are more similar to the adult game (Harwood et al., 2019). Specifically, playing on a shorter pitch increased the probability that children played short-pitch deliveries off the back foot rather than the front foot (a common feature of senior cricket). The implication is that children will learn to couple the action of playing a back foot shot when perceiving a short pitch delivery, and this will therefore augment development toward the adult game.

Unfortunately, however, few studies have examined the effect of scaled constraints on skill acquisition over a substantive period of time, with the intervention period often being short (e.g., 5 weeks). Nonetheless, these studies have revealed positive results regarding the effect of scaling on the performance outcome (e.g., sustaining a rally or hitting accurately) (Elliott, 1981; Farrow and Reid, 2010). Notably, Fitzpatrick et al. (2018) showed that children adapted to the constraints of the task over a period of 8 weeks. In this study, children were exposed to tennis tasks, including point-play akin to competition, but with the addition of specific constraints that aimed to shape behavior. For example, a recovery box location was positioned off center behind the court on the children’s forehand side and children were asked to return to this box after every point. The resultant behavior was an increase in backhands being performed. Hence, within the context of weekend junior sport, children will likely adapt to the constraints of the game by exploring and adopting solutions that generate success while satisfying the task constraints. Over time, and with enough repetitions, these solutions will stabilize and develop into *learnt* behavior. Clever manipulation of constraints can therefore promote the emergence of new behaviors as children adapt to new task constraints. Indeed, exposing children to a variety of constraints in a random manner can promote movement degeneracy (Lee et al., 2014) – the ability to functionally complete a task with different movement solutions (Seifert et al., 2016), which is a characteristic of expert performers (Seifert et al., 2013). We therefore expect children’s skill acquisition to be augmented when continuously exposed to environments that are appropriately modified.

## Future Directions

Evidence clearly shows that concept of scaling junior sport can have a positive influence on children’s sporting experience. However, despite our best efforts to unpack the design of scaled sport, there are a number of important questions that remain answered.

### Should the Junior Game Look Like the Adult Game?

An implicit assumption of the scaling sport argument is the idea that the junior game should resemble the adult game with respect to match-play characteristics and behaviors. We assume that the preservation of important characteristics of the adult or professional game, *via* appropriate manipulations, affords the acquisition of skills important for the development of expertise. For instance, Kachel et al. (2015) argued that the lower compression green ball was superior to the standard yellow ball because rally speed was closer to the professional game and that this would lead to players learning to play the game more like adults. While this may seem intuitive, even necessary at some point in a player’s journey toward the *adult* game, when and how this should happen remains uncertain. Put more bluntly, we do not actually know how closely the child’s game should mirror the informational or spatio-temporal constraints of the adult game to augment their development. A combination of prospective and longitudinal experimental designs is required to solve this issue (Farrow et al., 2018).

### Paradoxes in Our Thinking

Intuitively we assume that scaling should be based on one specific variable that reflects maturation, such as height. For example, Limpens et al. (2018) argued that the net height in tennis should be approximately 50% of children’s height given that the full-size net height is about 50% of the professional tennis player’s height. Significantly, this assumption implies that scaled environments should linearly progress toward adult environments. However, we know that skill acquisition is a non-linear process (Davids et al., 2008). Indeed, non-linear pedagogy, which is grounded in ecological dynamics, implies that skill acquisition is enhanced when the learning environment embraces non-linearity by promoting variability (Chow et al., 2011). This means that creativity in altering constraints can help to develop an adaptive movement system and reduce the risk of skill imbalances over time. The concept of non-linearity can be interpreted to suggest that scaling guidelines should in fact follow a non-linear path, however this then raises the question of what scaling should be based on? Understanding this question will help sport administrators set guidelines for junior sport so that children’s sport experience is maximized.

Another paradox in the scaling literature is the notion of repetition, skill, and injury (Reid et al., 2018). For example, modifying the ball and court in tennis was suggested to promote a positive learning experience due to the increase in repetitions when playing in such conditions. Intuitively, this is a positive outcome as more repetitions should lead to greater improvements in skill. However, taken to an extreme, could more repetitions also load musculoskeletal tissue in such a way that the likelihood of overuse injuries is increased? Sport scientists and medical professionals should collaborate to answer this question, as this will guide recommendations regarding volume of play in junior sport.

### Engagement, Motor Competence, and Sustained Participation?

Scaling sport allows children to experience more success (Buszard et al., 2014a,b), which appears to aid greater engagement (Farrow and Reid, 2010) and self-efficacy (Chase et al., 1994; Arias, 2012b). By designing environments that promote opportunity for success, children are more likely to have a heightened perception of their own ability. This is significant as perceived motor competence is considered a precursor to engaging in sport and physical activity, and greater engagement improves actual motor competence (Stodden et al., 2008). Importantly, a cyclical relationship exists between motor competence and physical activity levels. Children who are more competent with their motor skills are more likely to engage in physical activity in adolescence (Barnett et al., 2009; Lopes et al., 2011). Hence, it seems reasonable to think that scaled environments in junior sport will heighten children’s perception of their own ability, which will then lead to more participation in the sport, improved actual motor competence, and a greater likelihood of sustained participation.

## Conclusion

Sports administrators have a unique opportunity to nurture children’s weekly sporting experience by appropriately manipulating the rules and dimensions of the game. Equally important, however, are researchers and sport scientists to ensure that scaling is evidenced-based and grounded in theory. Indeed, it is evident that appropriate scaling can positively shape children’s skill development, but there are still many unknowns that need to be addressed. These include (1) whether the junior game should mirror the informational or spatio-temporal constraints of the adult game, (2) whether scaling should be guided by a variable describing physical maturation and therefore follow a linear progression despite our knowledge of non-linearity in skill acquisition, (3) whether a by-product of scaling is a greater risk of overuse injuries due to increased repetitions, (4) and whether scaling positively influences the cyclical relationship between competence and physical activity. Addressing these issues will likely require more sophisticated approaches than currently adopted in the scaling sport literature, but in doing so we will help maximize the potential of all children, irrespective of age, gender, or skill.

## Author Contributions

TB wrote the first draft of the manuscript. MR and DF edited subsequent drafts. All authors contributed to manuscript revision, and read and approved the submitted version.

### Conflict of Interest

MR is employed by Tennis Australia and TB receives research funding from Tennis Australia.

The remaining author declares that the research was conducted in the absence of any commercial or financial relationships that could be construed as a potential conflict of interest.
